# Case-Control Study of the Effects of Gut Microbiota Composition on Neurotransmitter Metabolic Pathways in Children With Attention Deficit Hyperactivity Disorder

**DOI:** 10.3389/fnins.2020.00127

**Published:** 2020-02-18

**Authors:** Lin Wan, Wen-Rong Ge, Shan Zhang, Yu-Lin Sun, Bin Wang, Guang Yang

**Affiliations:** ^1^The First Medical Center of the Chinese PLA General Hospital, Beijing, China; ^2^Beijing Friendship Hospital, Capital Medical University, Beijing, China

**Keywords:** attention deficit hyperactivity disorder, child, gastrointestinal microbiome, shotgun metagenomics sequencing, neurotransmitter

## Abstract

**Background:**

Attention-deficit/hyperactivity disorder (ADHD) is a neuropsychiatric condition that may be related to an imbalance of neural transmitters. The gut microbiota is the largest ecosystem in the human body, and the brain-gut axis theory proposes that the gut microbiome can affect brain function in multiple ways. The purpose of this study was to explore the gut microbiota in children with ADHD and assess the possible role of the gut microbiota in disease pathogenesis to open new avenues for ADHD treatment.

**Methods:**

A case-control design was used. We enrolled 17 children aged 6–12 years with ADHD who were treated in the Pediatric Outpatient Department of the First Medical Center of the Chinese PLA General Hospital from January to June, 2019. Seventeen children aged 6–12 years were selected as the healthy control (HC) group. Fecal samples of cases and controls were analyzed by shotgun metagenomics sequencing. Alpha diversity and the differences in the relative abundances of bacteria were compared between the two groups. Functional annotations were performed for the microbiota genes and metabolic pathways were analyzed using the Kyoto Encyclopedia of Genes and Genomes (KEGG).

**Results:**

There was no significant difference in the alpha diversity of gut microbiota between the ADHD and HC groups. Compared with HCs, *Faecalibacterium* and *Veillonellaceae* were significantly reduced in children with ADHD (*P* < 0.05), *Odoribacter* and *Enterococcus* were significantly increased [linear discriminant analysis (LDA) > 2]. At the species level, *Faecalibacterium prausnitzii*, *Lachnospiraceae bacterium*, and *Ruminococcus gnavus* were significantly reduced in the ADHD group (*P* < 0.05), while *Bacteroides caccae*, *Odoribacter splanchnicus*, *Paraprevotella xylaniphila*, and *Veillonella parvula* were increased (*P* < 0.05). Metabolic pathway analysis revealed significant between-group differences in the metabolic pathways of neurotransmitters (e.g., serotonin and dopamine) (*P* < 0.05).

**Conclusion:**

Composition differences of gut microbiota in subjects with ADHD may contribute to brain-gut axis alterations and affect neurotransmitter levels, which could contribute to ADHD symptoms.

## Introduction

Attention-deficit/hyperactivity disorder (ADHD) is a neuropsychiatric disorder that occurs most frequently in school-age children and is characterized as inattention with or without excessive impulsivity and hyperactivity (Abramov et al., 2019). Previous studies have reported that ADHD pathogenesis may be associated with dysregulation of neurotransmitters such as dopamine, serotonin (5-hydroxytryptamine, 5-HT), and norepinephrine (Magula et al., 2019; Stewart et al., 2019; Suzuki et al., 2019). Others have shown that the incidence of ADHD may have a certain degree of heritability, and genes related to dopamine, norepinephrine, and 5-HT transmission have been found to be abnormally expressed in children with ADHD (Banerjee and Nandagopal, 2015; Karmakar et al., 2017; Kim et al., 2018). Although various theories have been proposed, the pathogenetic mechanisms underlying ADHD have not been fully clarified, which limits the development of new treatments.

Gut microbiota alterations may be associated with neurological conditions including Alzheimer’s disease, epilepsy, and autism (Fan et al., 2019; Rude et al., 2019). Many researchers have proposed the existence of bidirectional regulation of the brain-gut axis, which involves gut microbiota metabolites that affect neurotransmitter levels, thereby influencing brain function (Melli et al., 2016; Khalil et al., 2019; Lacorte et al., 2019). In addition, nervous system activity can also impact gut microbiota composition. This bidirectional regulation is accomplished via complex neuroendocrine pathways (Khalil et al., 2019). The gut microbiota can adjust these pathways by regulating the levels of neurotransmitters and inflammatory factors and affecting the hypothalamic-pituitary-adrenal axis (Bermúdez-Humarán et al., 2019). Therefore, abnormal intestinal flora composition may lead to abnormal neurotransmitter secretion, which may promote the development of neuropsychiatric diseases.

We conducted a case-control study to analyze differences in intestinal flora composition between children with ADHD and healthy control (HC) children, explore ADHD pathogenesis, and investigate potential new treatments for ADHD.

## Materials and Methods

### Study Subjects

Seventeen children aged 6–12 (median 8 years) with ADHD were selected from the Pediatric Outpatient Department of the First Medical Center of the PLA General Hospital between January and June, 2019. The inclusion criteria were: (1) The Kiddie Schedule for Affective Disorders and Schizophrenia (K-SADS, Present and Lifetime Version scales) was used to diagnosis ADHD, and subjects met the diagnostic criteria for ADHD in the Diagnostic and Statistical Manual of Mental Disorders, Fifth Edition (DSM-5) (Ng et al., 2019) based on the opinion of an experienced child psychiatrist (GY or LW); (2) no history of respiratory or digestive tract infection within 1 month; (3) no use of probiotics within 1 month; (4) no history of digestive diseases or other chronic diseases; (5) body mass index (BMI) < 20 kg/m^2^ (because obesity could cause gut microbiota abnormalities) (Salah et al., 2019); and (6) no allergic diseases such as allergic rhinitis or asthma. Seventeen children from different families aged 6–12 years (median 8 years) were selected as the HC group in the same period. The inclusion criteria were the same except that there was no diagnosis of ADHD based on DSM-5 criteria by K-SADS. All of the participating children were born full-term with normal deliveries. Subjects were excluded if they were on a special diet (e.g., vegetarian). All parents of the participating children completed the Conners Parent Rating Scales (CPRS) to assess ADHD symptom severity and exclude subjects with depressive or anxiety symptoms. Participants maintained their regular dietary patterns for a week, and a food diary was recorded for participants from both groups during this period in order to exclude the potential influence of any changes in diet on the intestinal flora. Stool samples were collected at 8:00 am in the Pediatric Outpatient Department and stored in a sterile plastic cup at −80°C prior to testing.

The study was approved by the PLA General Hospital Ethics Committee (no. 2018-278). All subjects’ guardians were informed about the intentions of this study, and gave written informed consent was obtained in accordance with the Declaration of Helsinki.

### Sequencing and Analysis

#### DNA Sequencing

A total of 34 stool samples were collected from 17 ADHD patients and 17 age-matched HCs. We applied shotgun metagenomic sequencing to the whole genome of the microorganisms for each specimen. Bead beating was performed to rupture the bacteria, DNA was extracted with HiPure Stool DNA kits (Angen Biotech Co., Ltd., Guangzhou, China), and Qubit 4.0 software (Thermo Fisher Scientific, Waltham, MA, United States) was used for quality assessment. The library was prepared with a KAPA Hyper Prep Kit (KAPA Biosystems, Wilmington, MA, United States) and paired-end sequencing was performed on an Illumina NovaSeq platform (Illumina, San Diego, CA, United States) with a reading length of 150 bp (PE150).

### Species Abundance and Gene Function Annotations

All genome sequencing data were preprocessed by KneadData^1^ to screen out low-quality short frame sequences and chimeric sequences among the structural primer sequences (Bolger et al., 2014). Bowtie2 (Langmead and Salzberg, 2012) was then used to align the reads with the human genome for host sequence contamination removal. This was carried out with human reference genome hg19)^2^.

HUMAnN2 (version v0.11.2) was used to analyze the species abundance, gene function, and metabolic pathways related to the processed sequencing data (Franzosa et al., 2018). HUMAnN2 first used MetaPhlAn2 (version 2.7.7, Li et al., 2014) to match the sequence with the established core genes to quickly locate the species included in the microbiota. Sequences were then compared with the pan-genome of the identified species and mapped to corresponding phylogenetic levels. The abundance of genes or gene families, and metabolic pathways were analyzed at different phylogenetic levels of interest.

To determine the gene functional annotations, we employed the Bowtie2 (version 2.3.4.3) to map the sequences after removing low-quality sequences and host sequences, to Integrated Gene Catalog databases and Kyoto Encyclopedia of Genes and Genomes (KEGG). On this basis, gene abundance and alpha diversity indexes were calculated, which involves using the Shannon, Chao1, and Simpson indexes to calculate the entropy values of gene abundance. Euclidean distance was also computed as the measurement of beta diversity, followed by principal component analysis (PCA) and permutational multivariate analysis of variance (PERMANOVA). PCA was performed using ade4 package and PERMANOVA was carried out using vegan package (R version 3.5.3)^3^.

#### Bioinformatics Analysis

Chi-square tests were performed by SPSS 21.0 to compare sex differences between the ADHD and HC groups, and independent-sample *t*-tests were used to compare age, BMI, and CPRS scores. Wilcoxon tests were used by SPSS 21.0 to assess differences in species abundance and gene function between the ADHD and HC groups. The LDA effect size (LEfSe) method was used to determine the most differentially abundant taxa at the genus and species levels between the two groups.

## Results

### Comparison of Clinical Data Between the ADHD and HC Groups

A total of 17 ADHD children were included in this study, including 14 (82.3%) males and 3 (17.7%) females with a median age of 8 (25th and 75th percentiles: 7, 10) and a mean BMI of 16.1 ± 1.2 kg/m^2^. The 17 HCs included 13 (76.5%) males and 4 (23.5%) females with a median age of 8 (7, 9.5) and a mean BMI of 15.9 ± 1.1 kg/m^2^. There was no significant difference in the distributions of sex, age, or BMI between the two groups (*P* > 0.05). More children in the ADHD group (12, 70.5%) developed symptoms of constipation than in the HC group (2, 11.7%). The total CPRS scores were significantly different between the ADHD and HC groups (10.3 ± 4.2 vs. 2.2 ± 0.63, respectively; *P* < 0.05). There were no significant differences in the subscores for psychosomatic symptoms (0.56 ± 0.34 vs. 0.53 ± 0.41) or anxiety (0.42 ± 0.32 vs. 0.51 ± 0.35) (*P* > 0.05, Table 1).

**TABLE 1:** Descriptive data of the ADHD and HC groups.

	**ADHD (*n* = 17)**	**HC (*n* = 17)**	***P***
Sex, *n* (%)			0.671
Male	14(82.3%)	13(76.5%)	
Female	3(15%)	4(23.5%)	
Age, years; median (25th and 75th percentiles)	8(7,10)	8(7,9.5)	0.701
BMI, mean (SD)	16.1 (1.2)	15.9 (1.1)	0.652
Constipation, *n*(%)	12(70.5%)	2(11.7%)	<0.05
**ADHD symptom severity, mean (SD)**			
Total CPRS score	10.3 (4.2)	2.2 (0.63)	<0.05
Conduct problems	3.1 (1.46)	0.16 (0.27)	<0.05
Impulsive–hyperactivity	1.5 (0.59)	0.16 (0.22)	<0.05
Hyperactivity	3.4 (0.65)	0.05 (0.21)	<0.05
Learning problems	1.9 (0.57)	0.21 (0.34)	<0.05
Psychosomatic	0.56 (0.34)	0.53 (0.41)	0.452
Anxiety	0.42 (0.32)	0.51 (0.35)	0.523

*ADHD, attention-deficit/hyperactivity disorder; BMI, body mass index; CPRS, Conners Parent Rating Scales; HC, healthy control.*

### Analysis of Intestinal Flora Diversity

**FIGURE 1:**
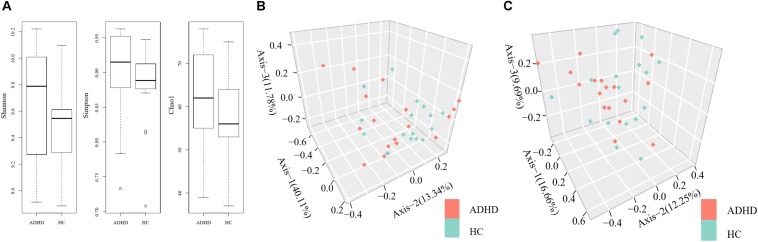
**(A)** Comparison of alpha diversity indexes between the ADHD and HC groups. **(B)** Permutational multivariate analysis of variance (PERMANOVA) of microbial communities of the participants at the genus level. **(C)** PERMANOVA of microbial communities of the participants at the species level.

The Shannon (9.67 ± 0.42 vs. 9.52 ± 0.25), Chao1 (61.5 ± 11.6 vs. 57.5 ± 9.8), and Simpson (0.89 ± 0.07 vs. 0.88 ± 0.06) indexes were calculated to assess the alpha diversity of fecal microbiota in the ADHD and HC groups. There were no significant differences in index values between the two groups (Figure 1A). At the genus level and the species level, PERMANOVA could not discriminate the ADHD from the HC group due to significant individual variation (Figures 1B,C).

### Analyses of Fecal Bacterial Community Abundance

**FIGURE 2:**
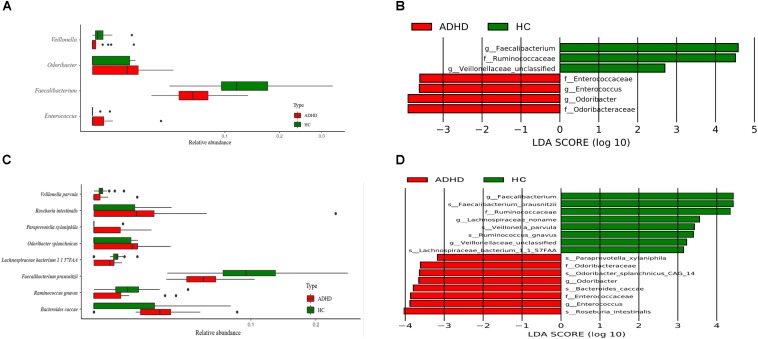
Comparison of different bacteria at the genus and species level between the ADHD and HC groups. **(A)** Wilcoxon test result at the genus level (*P* < 0.05). **(B)** LEfSe result at the genus level (LDA > 2). **(C)** Wilcoxon test result at the species level (*P* < 0.05). **(D)** LEfSe result at the species level (LDA > 2).

At the genus level, Wilcoxon tests showed that *Faecalibacterium* and *Veillonellaceae* were significantly reduced in the ADHD group, while *Odoribacter* was significantly higher (*P* < 0.05, Figure 2A). The LEfSe results also indicated that *Enterococcus* was significantly increased in the ADHD group (LDA > 2, Figure 2B).

At the species level, Wilcoxon tests showed that *Faecalibacterium prausnitzii*, *Lachnospiraceae bacterium*, and *Ruminococcus gnavus* were significantly decreased in the ADHD group, while *Bacteroides caccae*, *Odoribacter splanchnicus*, *Paraprevotella xylaniphila*, and *Veillonella parvula* were significantly increased (*P* < 0.05, Figure 2C). The results of LEfSe showed that *Odoribacteraceae* and *Enterococcaceae* were significantly increased in the ADHD group, while *Ruminococcaceae* was significantly decreased (LDA > 2, Figure 2D).

### KEGG Analysis of Metabolism

**FIGURE 3:**
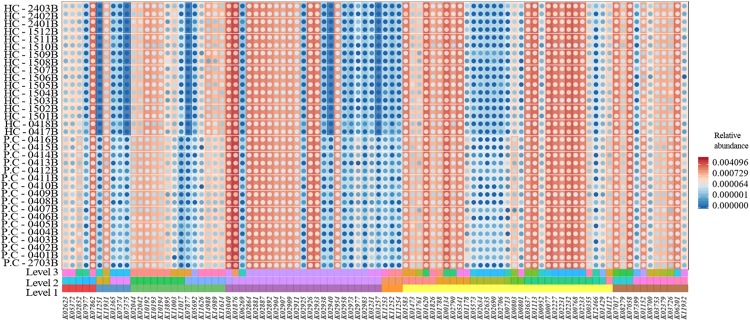
Comparison of Kyoto Encyclopedia of Genes and Genomes (KEGG) functional annotations between the ADHD and HC groups.

**FIGURE 4:**
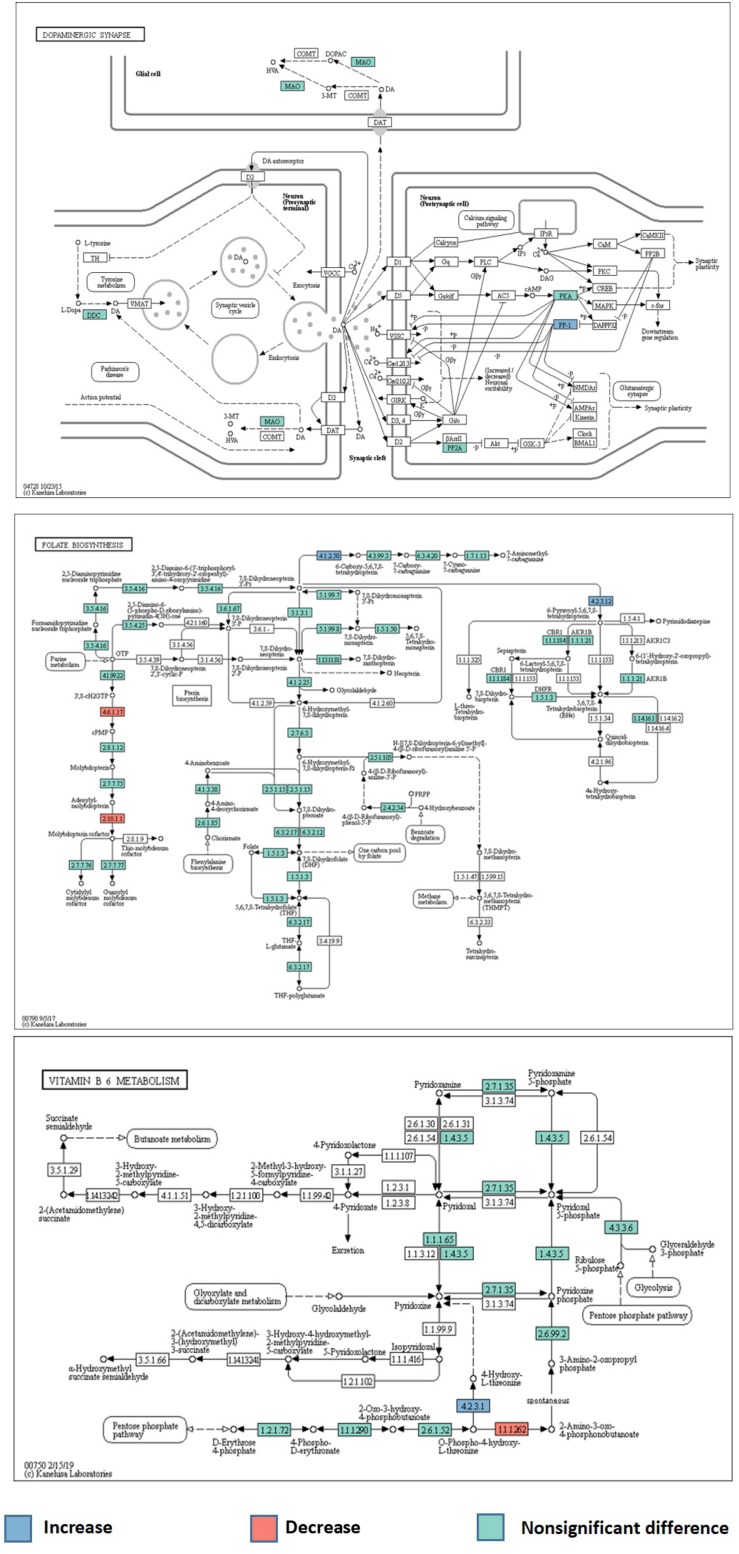
Abnormal metabolic pathways of neurotransmitters in the ADHD group.

A total of 6294 KEGG Orthology (KO) terms were used to annotate the genes. Wilcoxon tests showed 91 KOs that were significantly different between the two groups (*P* < 0.01, Figure 3). These included terms related to the neurotransmitter dopamine; the genes encoding the catalytic subunit of protein phosphatase-1 (PP1), threonine synthase, and 6-pyruvoyl-5,6,7,8-tetrahydropterin were significantly upregulated in the ADHD group, while the gene encoding 4-hydroxy threonine-4-phosphate dehydrogenase was significantly downregulated (*P* < 0.05, Figure 4).

## Discussion

The mammalian intestinal tract contains more than 100 trillion microorganisms; as the largest ecosystem in the body, it influences host physiological functions (Agus et al., 2018). The brain-gut axis theory proposes that there is a bidirectional regulatory mechanism between the intestinal flora and the brain. Children with ADHD may have abnormal neurotransmission, and the intestinal flora may regulate the level of neurotransmitters via complex neuroendocrine pathways (Richarte et al., 2018). A systematic review revealed two studies that assessed the correlation between ADHD and intestinal flora (Lacorte et al., 2019). Both employed 16S rRNA-sequencing technology and only analyzed the difference in gut microflora (Jiang et al., 2018; Prehn-Kristensen et al., 2018). We also applied shotgun metagenomic sequencing to the whole genomes of microorganisms for each specimen, and KEGG was used to analyze the metabolic pathways and identify possible pathogenetic mechanisms.

Similar to earlier reports (Jiang et al., 2018; Prehn-Kristensen et al., 2018), we found obvious differences in the gut microbiota of the ADHD and HC groups. Contrary to one study (Prehn-Kristensen et al., 2018), we found the alpha diversity of intestinal flora was not significantly different between groups, but subjects with ADHD had significantly lower levels of *Faecalibacterium*. However, unlike a previous report (Jiang et al., 2018), we also found that the ADHD group had significant decreases in *Veillonellaceae*, while *Enterococcus* and *Odoribacter* were significantly increased. At the species level, *F. prausnitzii*, *L. bacterium*, and *R. gnavus* were significantly reduced in the ADHD group, while *B. caccae*, *O. splanchnicus*, *P. xylaniphila*, and *V. parvula* were significantly increased. Additionally, we found that children with ADHD were more prone to constipation; consistent with our finding, previous studies have reported that the imbalance of intestinal flora is closely related to the occurrence of constipation (Huang et al., 2018; Wen et al., 2018; Wang L. et al., 2019).

The pathogenesis of ADHD remains unclear. One research group reported that abnormal levels of neurotransmitters are involved in the disease process (Kovács et al., 2019). Based on this theory, central nervous system (CNS) stimulants such as methylphenidate hydrochloride are widely used as first-line treatments for ADHD. The mechanism may involve inhibition of presynaptic reuptake of noradrenaline and dopamine; higher synaptic levels of these neurotransmitters may help to control symptoms, but clinical treatment effects vary among patients (Wigal et al., 2017).

Previous studies have shown that the gut microbiota could affect the brain-gut axis and contribute to the pathogenesis of neurological diseases including Parkinson’s, epilepsy, autism spectrum disorders, and tic disorders (Zhao et al., 2017; Kovács et al., 2019; Rude et al., 2019). Early intestinal flora establishment can affect nervous system development, resulting in anxiety behaviors and other mental health problems after maturity, and treatment of pregnant female rats with low-dose antibiotics has been shown to lead to an imbalance of intestinal flora, with subsequent alterations in the behavior of offspring (Borre et al., 2014; Leclercq et al., 2017; Zhao et al., 2017). In another study where gut bacteria from patients with schizophrenia were transplanted into germ-free mice, the mice developed psychotic symptoms due to altered regulation of the glutamine-glutamate-gamma-aminobutyric acid pathway by the transplanted gut bacteria (Zheng et al., 2019). Similarly, transplantation of intestinal flora from patients with Parkinson’s disease into a germ-free Parkinson’s disease mouse model significantly increased motor symptoms of the mice, which were ameliorated by antibiotic treatment (Sampson et al., 2016).

According to the results of our experiment, we speculated that the abnormality of intestinal flora might be one of the bases of the onset of ADHD, combined with previous studies, we proposed the following conjecture about its mechanism of action. In this study, children with ADHD exhibited a reduction of *Faecalibacterium*. This has been observed in both animal and human studies and has been implicated in various allergic diseases such as asthma, eczema, and allergic rhinitis (Penders et al., 2007; Arrieta et al., 2015; Melli et al., 2016). In clinical practice, atopic children have a 30–50% increased risk of ADHD (Schans et al., 2017). We therefore speculate that the reduction of this bacterial genus may generate allergies via the brain-gut axis by affecting neurotransmitter release and inducing the pathogenesis of ADHD. One study reported that ADHD was more likely to be induced by diets high in fat, protein, and sugar, which also decrease *Faecalibacterium* levels (Howard et al., 2011). *Faecalibacterium* may exert anti-inflammatory effects, and the abnormal levels may lead to higher expression of inflammatory factors (Qiu et al., 2013; Quévrain et al., 2016). Notably, children with ADHD have significantly higher levels of inflammatory cytokines than normal children (Mitchell and Goldstein, 2014). Inflammatory cytokines can cross the blood–brain barrier (BBB) and affect nervous system development and brain function (Wong et al., 2016). We therefore hypothesized that *Faecalibacterium* dysregulation may cause changes in inflammatory cytokine levels and participate in ADHD pathogenesis.

We also found that the proportion of *Enterococcus* was significantly increased in the ADHD group, and *Enterococcus* has been reported to be closely related to neurotransmitter release. One study demonstrated that *Enterococcus* abundance is significantly increased in mice lacking the 5-HT transporter (Singhal et al., 2019); deficiency of this transporter can lead to decreased 5-HT levels, which is related to ADHD onset (Wang et al., 2018). Interestingly, a study showed *Enterococcus* could lead to excessive intestinal conversion of levodopa (the first-line treatment for Parkinson’s disease) into dopamine, however, peripheral dopamine cannot penetrate the BBB to enter the CNS, thus reducing the effectiveness of levodopa (Maini Rekdal et al., 2019). Furthermore, the abnormal increase in *Enterococcus* could also cause excessive activation of tyrosine decarboxylase, which increases the decarboxylation of tyrosine and phenylalanine in the gastrointestinal tract, leading to decreased levels in the CNS and subsequent low levels of levodopa (the drug precursor of dopamine) (Maini Rekdal et al., 2019). Both of these pathways can affect the concentration of dopamine in the CNS, which may aggravate Parkinson’s symptoms (Maini Rekdal et al., 2019). Previous studies have shown that ADHD onset is related to decreased CNS levels of dopamine (Roncero and Álvarez, 2014; Ledonne and Mercuri, 2017). As above, we speculate that the observed increase in *Enterococcus* may lower intracranial dopamine and contribute to the development of ADHD. In addition, our observation of a higher proportion of *Odoribacter* in subjects with ADHD is similar to the results of a previous study that found higher *Odoribacter* levels in individuals with pediatric acute-onset neuropsychiatric syndrome (PAN) and pediatric autoimmune neuropsychiatric disorders associated with streptococcal infections (PANDAS) (Quagliariello et al., 2018). Additionally, Phylogenetic Investigation of Communities by Reconstruction of Unobserved States (PICRUSt) analysis of this study showed that the dopamine metabolic pathway was significantly reduced in PAN and PANDAS (Quagliariello et al., 2018). *Odoribacter* may cause abnormalities in dopamine metabolism that contribute to ADHD. Previous studies (Quagliariello et al., 2018; Maini Rekdal et al., 2019) found that abnormal *Enterococcus* and *Odoribacter* levels were associated with dysregulated neurotransmitter production. Abnormal levels of these bacteria were also found in our study, suggesting a role in the development of ADHD.

Finally, we performed KEGG analysis to determine the gene functional annotations and abnormalities in metabolic pathways, to verify the speculation of the role of gut microbiota in the pathogenesis of ADHD. Reduced dopamine levels in the CNS may contribute to ADHD pathogenesis. We identified differences in the dopaminergic synaptic pathways between the ADHD and HC groups; the gene encoding PP1 catalytic subunit was significantly upregulated, which was considered to increase synaptic sodium ion flux. Dopamine receptors are transmembrane sodium/chloride-dependent transporters that belong to the family of transporters of norepinephrine, 5-HT, and dopamine, and are referred to as neurotransmitter: sodium symporters (NSS) (Navratna and Gouaux, 2019). Prolonged sodium-related signal transduction results in the excessive activation of NSS. Metabolic pathway alterations may cause abnormal neurotransmitter transport and reduce their concentrations in the CNS, which could contribute to ADHD. Numerous studies have reported that vitamin B6 plays a key role in nervous system development and neurotransmitter production. A randomized controlled trial of 216 children with ADHD and 216 healthy children found lower vitamin B6 levels in children with ADHD (Wang L.J. et al., 2019). In line with this finding, KEGG analysis indicated abnormalities in the metabolic pathway of vitamin B6 in the ADHD group. The genes encoding 4-hydroxy threonine-4-phosphate dehydrogenase and threonine synthase were significantly downregulated and upregulated, respectively, which could lead to abnormal levels of pyridoxal 5′-phosphate, which is an important coenzyme of aromatic amino acid decarboxylase (AADC) (Montioli et al., 2019). AADC is a key enzyme of dopamine metabolism that converts levodopa into dopamine in the CNS (Baek et al., 2018). A decrease in its activity could lead to the reduction of dopamine concentrations, which could contribute to ADHD onset. In the folate metabolic pathway, a significant upregulation of the gene encoding 6-pyruvoyl-5,6,7,8-tetrahydropterin could promote the generation of tetrahydrobiopterin (BH4). However, tryptophan hydroxylase is a rate-limiting enzyme that catalyzes 5-HT synthesis, with oxygen and BH4 as substrates (Opladen et al., 2016; Scotton et al., 2019). Upregulation of the gene encoding 6-pyruvoyl-5,6,7,8-tetrahydropterin may lead to the conversion of excessive tryptophan into 5-HT in the intestinal tract, and 5-HT has difficulty crossing the BBB, resulting in decreased CNS 5-HT concentrations, which may contribute to ADHD.

There are several limitations to this study. First, our sample size was relatively small. Second, we did not perform transplantation of intestinal flora to confirm that gut microbiota composition affects ADHD symptoms.

## Conclusion

In summary, our results demonstrate that gut microbiota alterations occur in children with ADHD, which may contribute to abnormal metabolism of neurotransmitters. We cautiously speculated that the abnormal intestinal flora might be one of contributing factors of ADHD, the underlying mechanism may be related to changes in microbial functions that affect the function of the neuroendocrine system, leading to reduced levels of 5-HT and dopamine in the CNS, and ultimately to ADHD. Further studies should be carried out to investigate the CNS levels of dopamine and 5-HT, and animal studies are needed for functional verification.

## Data Availability Statement

The raw data supporting the conclusions of this article will be made available by the authors, without undue reservation, to any qualified researcher.

## Ethics Statement

The studies involving human participants were reviewed and approved by the PLA General Hospital Ethics Committee. Written informed consent to participate in this study was provided by the participants’ legal guardian/next of kin.

## Author Contributions

LW and W-RG contributed equally to the manuscript. LW and GY contributed to the study conception and design. W-RG, SZ, Y-LS, BW, and LW organized the database. W-RG, Y-LS, SZ, LW, and GY performed the statistical analysis. W-RG, LW, and GY wrote the first draft of the manuscript. All authors wrote sections of the manuscript, contributed to the manuscript revision, and read and approved the submitted version.

## Conflict of Interest

The authors declare that the research was conducted in the absence of any commercial or financial relationships that could be construed as a potential conflict of interest.

## Footnotes

1 https://bitbucket.org/biobakery/kneaddata

2 http://hgdownload.soe.ucsc.edu/goldenPath/hg19/bigZips/hg19.fa.gz

3 https://www.r-project.org/
